# Circulating Vitamin E Levels and Risk of Coronary Artery Disease and Myocardial Infarction: A Mendelian Randomization Study

**DOI:** 10.3390/nu11092153

**Published:** 2019-09-09

**Authors:** Tao Wang, Lin Xu

**Affiliations:** 1 School of Public Health, Sun Yat-sen University, Guangzhou 510080, China; 2 School of Public Health, the University of Hong Kong, Hong Kong, China

**Keywords:** vitamin E, cardiovascular disease, mendelian randomization

## Abstract

Observational studies have reported a cardioprotective effect of vitamin E whereas intervention trials failed to confirm its beneficial effects, and even some reported adverse effects of vitamin E supplements on coronary artery disease (CAD). To clarify, we conducted a two-sample mendelian randomization study to investigate causal association of vitamin E with the risk of CAD. Three single nucleotide polymorphisms (SNPs) identified in a genome-wide analysis study including 7781 individuals of European descent, rs964184, rs2108622, and rs11057830 were used as the genetic instruments for vitamin E. Data for CAD/myocardial infarction (MI) were available from Coronary ARtery DIsease Genome wide Replication and Meta-analysis (CARDIoGRAM) plus The Coronary Artery Disease (C4D) Genetics consortium. The effect of each SNP on CAD/myocardial infarction (MI) was weighted by its effect on serum vitamin E (mg/L), and results were pooled to give a summary estimates for the effect of increased vitamin E on risk of CAD/MI. Based on 3 SNPs each 1 mg/L increase in vitamin E was significantly associated with CAD (odds ratio (OR) 1.05, 95% confidence interval (CI) 1.03–1.06), MI (OR 1.04, 95% CI 1.03–1.05), elevated low-density lipoprotein cholesterol (0.021 standard deviations (SD), 95% CI 0.016, 0.027), triglycerides (0.026 SD, 95% CI 0.021, 0.031), and total cholesterol (0.043 SD, 95% CI 0.038, 0.048) and lower levels of high-density lipoprotein cholesterol (−0.019 SD 95% CI −0.024, −0.014). Our findings indicate that higher vitamin E may increase the risk of CAD/MI and the safety and efficacy of vitamin E supplementation use should be reevaluated.

## 1. Introduction

Vitamin E is a fat-soluble antioxidant vitamin that protects lipids from peroxidation in vitro [1]. Increasing evidence supports a central role for lipid oxidation in the development and progression of atherosclerosis [2,3]. Therefore, vitamin E has been postulated to attenuate the process of atherosclerosis and reduce the risk of cardiovascular disease. Several observational studies showed that higher vitamin E intake from dietary sources or supplements was associated with a lower risk of cardiovascular disease (CVD) [4,5,6,7,8]. Dietary or supplemental vitamin E intake as measured by food frequency questionnaires may not be well correlated with serum vitamin E concentrations, or reflect the lifelong vitamin E exposure. Moreover, the protective effects of vitamin E against CVD might be due to the combination of various antioxidants and nutritional substances contained in daily food. Some studies examining the association of serum vitamin E with the risk of CVD showed inconsistent results [9,10,11,12,13,14], with some study showing a cardioprotective effect [13,14], while some reporting vitamin E has no effect on CVD [9,10,11,12]. Reverse causation could be possible in the studies above since antioxidant may be depleted due to oxidative stress in the process of atherosclerosis. Randomized control trials (RCTs) are considered the gold standard to determine short- or moderate-term effects of a given intervention. The majority of vitamin E intervention trials and meta-analyses of RCTs, however, have failed to confirm its beneficial effects on CVD [15,16,17,18,19,20]. Notably, vitamin E supplementation was shown to increase the risk of heart failure in two RCTs [21,22] and the risk of angina pectoris in a meta-analysis [20]. Further, three meta-analyses of vitamin E supplementation trials showed an increased risk of all-cause mortality [23,24,25]. Hence, to date, it remains unclear whether vitamin E has beneficial or harmful effects on the risk of CVD. 

As residual confounding and reverse causation are inevitable in observational studies, and long-term large-scale RCTs might not be feasible and ethical, we therefore used Mendelian randomization (MR) as an alternative method to make stronger causal inference. MR studies make use of genetic variants as instrumental variables (IVs) to investigate causal effects of exposure factors on health outcomes. Since genetic variants are randomly allocated at conception, MR studies may provide genetic randomization analogous to the randomization in RCTs and are less biased by confounding and reverse causation [26].

Here, we used genetic variants (i.e., single nucleotide polymorphisms (SNPs)) identified in a genome wide association study (GWAS) [27] to examine causal association of vitamin E levels with the risk of coronary artery disease (CAD) and cardiovascular risk factors.

## 2. Materials and Methods

### 2.1. Data Sources

#### 2.1.1. Vitamin E Levels

From a GWAS including 7781 individuals of European descent, we obtained SNPs independently associated with circulating vitamin E levels with genome-wide significance (*p* < 5.0 × 10^−8^) [27]. Briefly, the GWAS of circulating alpha-tocopherol concentrations were conducted in the Alpha-Tocopherol, Beta-Carotene Cancer Prevention Study cohort (*n* = 4014) and replicated these findings in a combined meta-analysis with the Prostate, Lung, Colorectal, and Ovarian Cancer Screening Trial (PLCO) Study (*n* = 992) and the Nurses’ Health Study (NHS) (*n* = 2775). Three genetic variants associated with circulating alpha-tocopherol concentrations were identified, including rs964184 (*p* = 7.8 × 10^−12^), rs2108622 (*p* = 1.4 × 10^−10^), and rs11057830 (*p* = 8.2 × 10^−9^). The pleiotropic effects of these three SNPs were checked from Ensembl (Homo sapiens-phenotype) (http://grch37.ensembl.org/Homo_sapiens/Info/Index), a comprehensive genotype to phenotype cross-reference (Table S1).

#### 2.1.2. Coronary Artery Disease and Its Risk Factors

Association of SNPs with the phenotypes were extracted from publicly available consortia. Data on CAD/myocardial infarction (MI) have been contributed by CARDIoGRAMplusC4D (Coronary ARtery DIsease Genome wide Replication and Meta-analysis (CARDIoGRAM) plus The Coronary Artery Disease (C4D) Genetics) investigators and have been downloaded from www.CARDIOGRAMPLUSC4D.ORG [28]. The summary data on the gene-CAD association were obtained from the CARDIoGRAMplusC4D 1000 Genomes-based GWAS, a meta-analysis of GWAS studies of mainly European, South Asian, and East Asian, descent imputed using the 1000 Genomes phase 1 v3 training set with 38 million variants [29]. The study interrogated 9.4 million variants and involved 60,801 CAD cases and 123,504 controls, and 43,676 MI cases and 128,199 controls [29]. Data on T2DM was contributed by the DIAbetes Genetics Replication And Meta-analysis (DIAGRAM, http://diagram-consortium.org/downloads.html), which includes 12,171 cases and 56,862 controls in Stage 1 GWAS [30] and 26,488 cases and 83,964controls in the Trans-ethnic GWAS meta-analysis [31].

Genetic associations with high density lipoprotein (HDL) cholesterol, low density lipoprotein (LDL) cholesterol, triglycerides, and total cholesterol in 188,577 people have been contributed by Global Lipids Genetics Consortium (GLGC) investigators and have been downloaded from http://csg.sph.umich.edu/abecasis/public/lipids2013/ [32]. Genetic associations with fasting insulin (*n* = 38,238) and fasting glucose (*n* = 46,186) have been contributed by Meta-Analyses of Glucose and Insulin-related traits Consortium (MAGIC) investigators and have been downloaded from http://www.magicinvestigators.org/, which relates to people of European ancestry without diabetes [33,34,35].

### 2.2. Statistical Analysis

SNP-specific Wald estimates (ratio of SNP on outcome to SNP on alpha-tocopherol) of the effect of vitamin E levels on each outcome (Table S2) were combined using inverse-variance weighted (IVW) method giving an odds ratio (OR) for CAD and MI, and beta coefficients for the other outcomes with 95% confidence interval (CI), based on the following formulas [36]:

(1) $${\hat{\beta }}_{IVW}=\frac{\sum_{k\;=\;1}^{K\;}E_{k}D_{k}\sigma_{D_{k}}^{–2}}{\sum_{k\;=\;1}^{K}E_{k}^{2}\sigma_{D_{k}}^{–2}},$$

(2) $$SE_{{\hat{\beta }}_{IVW}}=\sqrt{\frac{1}{\sum_{k\;=\;1}^{K}E_{k}^{2}\sigma_{D_{k}}^{–2}}},$$

where *E*_k_ is the mean change in exposure level (vitamin E) per additional effect allele of SNP k and *D*_k_ is the mean change in disease outcomes (e.g., log odds of CAD or levels of other CVD risk factors) per additional effect allele of SNP k with standard error $\sigma_{D_{k}}$. The weakness of the instruments was evaluated using the first-stage F-statistics calculated by F $=\frac{n\;-\;k\;-1}{k}\frac{R^{2}}{\left(1\;-\;R^{2}\right)}$, where $R^{2}$ denote the proportion of exposure variability explained by genotype, k is the number of genetic instruments, and n is a sample size of the first stage [35]. As the rs964184 was also associated with cholesterol and triglycerides and may introduce pleiotropic effect, we excluded it from the MR analysis as a sensitivity analysis. 

## 3. Results

The first-stage F-statistics for the IVs including these 3 SNPs was 48. Table 1 shows that these three genetic variants served as IVs in the current MR analysis are located in different chromosomes and not in linkage disequilibrium.

Table 2 shows that the ORs per 1 mg/L increase in genetically predicted vitamin E levels were 1.05 (95% CI 1.032, 1.057) for CAD and 1.04 (95% CI 1.030, 1.058) for MI. One mg/L higher vitamin E concentration was associated with elevated LDL-cholesterol (0.021 SD (1 SD = 38.7 mg/dL), 95% CI 0.016, 0.027), triglycerides (0.026 SD (1 SD = 90.7 mg/dL), 95% CI 0.021, 0.031), and total cholesterol (0.043 SD (1 SD = 41.8 mg/dL), 95% CI 0.038, 0.048) and lower levels of HDL-cholesterol (−0.019 SD (1 SD = 15.5 mg/dL), 95% CI −0.024, −0.014). However, no association was found for type 2 diabetes and fasting glucose.

Results were similar in the sensitivity analyses based on 2 SNPs (rs2108622, rs11057830), although the association for HDL-cholesterol was not statistically significant (−0.004 SD, 95% CI −0.009, 0.002) (Table 3). 

## 4. Discussion

In the current mendelian randomization analysis, we found that higher vitamin E levels are causally associated with increased risks of CAD and MI. In addition, vitamin E levels were also significantly associated with CAD risk factors including LDL-cholesterol, total cholesterol and triglycerides. No evidence for a causal effect of vitamin E on type 2 diabetes and fasting glucose was found.

Previously observational studies mostly showed an inverse association between vitamin E and the risk of CVD [7,8,13,14]. However, this association may be explained by residual confounding or reverse causality or both. In contrast, evidence from most RCTs did not support the use of vitamin E supplementation for the primary or secondary prevention of CVD [15,16,17,18,37,38,39], although there were two RCTs reporting a beneficial effect of vitamin E supplementation on CVD outcomes [40,41]. This discrepancy may be due to the dose of supplementation use (from 30 mg to 800 mg), follow-up time (from 1.5 years to 7 years), subtypes of vitamin E (natural or synthetic), and participants characteristics (apparently healthy participants or participants at high risk). Notably, some RCTs [21,22] and meta-analyses of RCTs [20,42] showed that vitamin E supplementation intake increased the risks of heart failure, angina pectoris, or hemorrhagic stroke. The Heart Outcomes Prevention Evaluation (HOPE) trial and extended study (The Ongoing Outcomes) (HOPE-TOO) suggested that in patients at high risk of CVD, vitamin E supplementation of 400 IU/d significantly increased the risk of heart failure during a median follow-up period of 7.0 years (RR 1.13, 95% CI 1.01, 1.26) [21]. Moreover, a meta-analysis of fifty RCTs including 294,478 participants showed that, although vitamin E supplementation group had a lower risk of MI, the beneficial effect was not evident in subgroup analysis excluding trials funded by pharmaceutical industry (RR 0.79, 95% CI 0.53, 1.17) [20]. In addition, vitamin E supplementation appeared to be marginally associated with an increased risk of angina pectoris, although the result was not statistically significant (RR 1.15, 95% CI 0.99, 1.33) [20]. The present MR analysis was in accordance with some previous RCTs by showing that lifelong higher vitamin E exposure was associated with higher risks of CAD and MI, and such associations could be causal. As the genotypes are fixed at conception, the genetically determined vitamin E levels may be more likely to reflect a lifetime exposure, whereas RCTs are usually conducted within a short time period in a relatively small size and thus may be insufficient to detect the potentially unfavorable effect of vitamin E on CVD [43].

Our study also showed that vitamin E was causally associated with higher levels of triglycerides, LDL- and total-cholesterol, and lower levels of HDL-cholesterol, which may partly account for the adverse effects of Vitamin E on CAD. Some other possible mechanisms have been proposed. For example, it is reported that alpha-tocopherol may become a pro-oxidant in an oxidative environment, thereby depressing myocardial function [44]. There is also evidence that alpha-tocopherol may suppress other fat-soluble antioxidants, such as gamma-tocopherol, disrupt the natural balance of antioxidant systems, and increase vulnerability to oxidative damage [45]. 

There are some strengths in the current study. First, the use of MR analysis may minimize reverse causality and confounding from non-genetic factors. Second, as the three genetic variants are located in different chromosome, the potential gene-gene influence on the estimates (i.e., linkage disequilibrium) may be unlikely. Third, as in two-sample MR, data on the genetic variants-exposure association and the genetic variants-outcomes association can be obtained from different sample of individuals, genetic associations with the exposure/outcomes can be obtained from large consortia, which greatly increases statistical power to detect small effects in complex phenotypes [46]. Fourth, as we used genetic instruments strongly associated with vitamin E, as suggested by an acceptable F-statistic value, it is unlikely that our results were biased by weak instruments. Fifth, our findings are unlikely confounded by population stratification because the analyses included populations of mainly European ancestry. Finally, MR studies are susceptible to pleiotropic effects (vitamin E levels related genetic variants also associated with other phenotypes that might cause CAD). In a sensitivity analysis, we excluded a genetic variant (rs946184) from the MR analysis to avoid pleiotropic bias and results remained the same. Thus the positive association between vitamin E and CAD might not be explained by pleiotropic effects.

Our study also has several limitations. First, vitamin E exists as two major classes including tocopherols and tocotrienols, both of which further subcategorized into alpha, beta, gamma, and delta forms. Alpha-tocopherol is the most biologically active form of vitamin E and almost exclusively used in vitamin E intervention trials. However, it is reported that gamma-tocopherol may be important to human health and that it possesses unique features that distinguish it from alpha-tocopherol [45]. In the present study, we only selected alpha-tocopherol levels to represent circulating vitamin E levels. Hence, further studies accounting for other vitamin E forms, such as gamma-tocopherol, are warranted. Second, due to the use of aggregated genome-wide data, whether the effect of vitamin E on CAD varies by health/disease status, age or sex cannot be examined. Note that previous observational studies focused on general population mostly reported a protective effect of vitamin E on CVD, whereas some RCTs focused on individuals at high risk of CVD or with pre-existing CVD showed harmful effects on heart failure. Third, as other MR studies, our study cannot rule out the effect of canalization (i.e., dilution of the gene-exposure association) and thus the estimate may be inflated. The use of multiple SNPs as IV may more or less compensate this influence. Fourth, we found that one vitamin E related SNP (rs964184) was also associated with lipids and thus pleiotropy may be a concern. However, as vitamin E may influence coronary artery disease through its effects on lipids, the pleiotropy effect, if any, tends to be vertical. Vertical pleiotropy is generally not problematic in MR. Moreover, we also excluded this SNP in sensitivity analyses and found that the results remained. Finally, given the small number of SNPs employed in this study, further MR analyses using more functionally relevant SNPs from up-to-date GWASs are needed to confirm results of the current study.

## 5. Conclusions

In conclusion, present MR analysis showed that higher vitamin E was causally associated with high risks of CAD and MI. Our findings, along with previous RCTs regarding the unfavorable effects of vitamin E supplementation, suggest that the use of vitamin E supplementation for primary and secondary prevention of cardiovascular disease should be reevaluated substantially in terms of its safety and efficacy.

## Figures and Tables

**Table 1:** Characteristics of three SNPs in previously published vitamin E GWAS data set ^a^.

SNP	Chromosome	Nearby Gene	Effect Allele ^b^	Other Allele	Effect (Beta) ^c^	SE	*p*-Value	EAF
rs11057830	12	SCARB1	A	G	0.03	0.01	8.2 × 10^−9^	0.15
rs2108622	19	CYP4F2	T	C	0.03	0.01	1.4 × 10^−10^	0.21
rs964184	11	BUD13/ZNF259/APOA5	G	C	0.04	0.01	7.8 × 10^−12^	0.15

SNPs, single-nucleotide polymorphisms; SCARB1, scavenger receptor class B member 1; CYP4F2, cytochrome p450, family 4, subfamily F, polypeptide 2; BUD13, (yeast) budding site selection protein 13; ZNF259, zinc finger protein 259; APOA5, apolipoproteins A5; SE, standard error; EAF, effect allele frequency; ^a^ Information of these SNPs was obtained from a published GWAS on 7781 individuals of European descent [27]. ^b^ Vitamin E increasing allele. ^c^ Increase in vitamin E levels (mg/L) per effect allele.

**Table 2:** MR estimates of causality between vitamin E levels (mg/L) and cardiovascular risk factors, diabetes, and coronary artery disease based on 3 SNPs using fixed or random IVW model according heterogeneity test.

	Consortia	OR	95% CI	*p*-Value	IVW (Fixed or Random Effect Model)	Heterogeneity	
						I^2^	*p*-Value
Coronary artery disease	CARDIoGRAMplusC4D (*n* = 60,801 cases and 123,504 controls)	1.05	1.032 to 1.057	<0.001	Fixed	0	0.98
Myocardial infarction	CARDIoGRAMplusC4D (*n* = 43,676 cases and 128,199 controls)	1.04	1.03 to 1.058	<0.001	Fixed	0	0.54
Type 2 diabetes	DIAGRAM (*n* = 12,171 cases and 56,862 controls)	1.00	0.977 to 1.027	0.89	Fixed	47%	0.15
		Beta	95% CI	*p*-value			
LDL-C, SD (1 SD = 38.7 mg/dL)	Global Lipids Genetics Consortium (*n* = 188,577)	0.021	0.016 to 0.027	<0.001	Random	99%	<0.001
HDL-C, SD (1 SD = 15.5 mg/dL)	Global Lipids Genetics Consortium (*n* = 188,577)	−0.019	−0.024 to −0.014	<0.001	Random	98%	<0.001
TC, SD (1 SD = 41.8 mg/dL)	Global Lipids Genetics Consortium (*n* = 188,577)	0.026	0.021 to 0.031	<0.001	Random	99%	<0.001
TG, SD (1 SD = 90.7 mg/dL)	Global Lipids Genetics Consortium (*n* = 188,577)	0.043	0.038 to 0.048	<0.001	Random	99%	<0.001
Fasting glucose, mmol/L	Meta-Analyses of Glucose and Insulin-related traits Consortium (*n* = 46,186)	0.003	−0.003 to 0.008	0.98	Fixed	0	0.38

MR, Mendelian randomization; OR, Odds ratio; CI, confidential interval; IVW, inverse-variance weighted; I^2^, statistics indicating heterogeneity between estimates from multiple instrumental variables; LDL-C, low-density lipoprotein cholesterol; HDL-C, high-density lipoprotein cholesterol; TG, triglycerides; TC, total cholesterol.

**Table 3:** MR estimates of causality between vitamin E levels (mg/L) and cardiovascular risk factors, diabetes and coronary artery disease, excluding rs964184 from analysis.

	**OR**	**95% CI**	***p*-Value**
Coronary artery disease	1.04	1.028–1.059	<0.001
Myocardial infarction	1.04	1.027–1.06	<0.001
Type 2 diabetes	0.98	0.95–1.015	0.31
	**Beta**	**95% CI**	***p*-Value**
LDL-C, SD (1 SD = 38.7 mg/dL)	0.011	0.005–0.016	<0.001
HDL-C, SD (1 SD = 15.5 mg/dL)	−0.004	−0.009–0.002	0.20
TC, SD (1 SD = 41.8 mg/dL)	0.01	0.004–0.016	0.001
TG, SD (1 SD = 90.7 mg/dL)	0.009	0.003–0.014	0.002
Fasting glucose, mmol/L	0.001	−0.006–0.007	0.85

MR, Mendelian randomization; OR, Odds ratio; CI, confidential interval; LDL-C, low-density lipoprotein cholesterol; HDL-C, high-density lipoprotein cholesterol; TG, triglycerides; TC, total cholesterol.

## Supplementary Materials

The following are available online at https://www.mdpi.com/2072-6643/11/9/2153/s1, Table S1: Related traits of three SNPs from Ensembl search, Table S2: Associations of three SNPs with CAD/MI and their risk factors.
