# Supplementary material for: Circulating Vitamin E Levels and Risk of Coronary Artery Disease and Myocardial Infarction: A Mendelian Randomization Study
Source: Nutrients. 2019 Sep 9;11(9):2153. doi: 10.3390/nu11092153 (PMC6770080; doi:10.3390/nu11092153)
Supplement: Supplementary file 1 [file nutrients-11-02153-s001.pdf]

Table S1. Related traits of three SNPs from Ensembl search.

SNP	Chromosome	Nearby Gene	Effect Allele	Other Allele	Related Traits
rs11057830	12	SCARB1	A	G	1. Vitamin E levels<br>2. Lipoprotein phospholipase A2 activity in cardiovascular disease<br>3. coronary artery disease
rs2108622	19	CYP4F2	T	C	1. Vitamin E levels<br>2. Acenocoumarol maintenance dosage<br>3. Warfarin maintenance dose<br>4. Circulating phylloquinone levels
rs964184	11	BUD13/ZNF259/APOA5	G	C	1. Vitamin E levels<br>2. LDL cholesterol<br>3. HDL cholesterol<br>4. Hypertriglyceridemia<br>5. Cholesterol, total<br>6. Triglyceride levels<br>7. Blood protein levels<br>8. Very long-chain saturated fatty acid levels (fatty acid 20:0)<br>9. Very low-density lipoprotein cholesterol levels<br>10. Red cell distribution width

Web of Ensembl (Homo sapiens-phenotype): http://grch37.ensembl.org/Homo_sapiens/Info/Index.

Table S2. Associations of three SNPs with CAD/MI and their risk factors.

SNP	Effect Allele	Other Allele	CAD		MI		T2D		HDL-C, SD		LDL-C, SD		TC, SD		TG, SD		FG, mmol/L	
			**beta**	**se**	**beta**	**se**	**beta**	**se**	**beta**	**se**	**beta**	**se**	**beta**	**se**	**beta**	**se**	**beta**	**se**
rs11057830	A	G	0.046	0.01	0.03	0.01	-0.01	0.03	-0.018	0.005	0.025	0.006	0.022	0.005	0.022	0.005	0.005	0.006
rs2108622	T	C	0.046	0.01	0.05	0.01	-0.02	0.02	0.003	0.004	0.004	0.004	0.005	0.004	0.003	0.004	-0.001	0.004
rs964184	G	C	0.050	0.01	0.05	0.01	0.04	0.02	-0.107	0.007	0.086	0.008	0.121	0.008	0.234	0.007	0.008	0.005

SNPs, single-nucleotide polymorphisms; SD, standard deviation; SE, standard error; CAD, coronary artery disease; MI, myocardial infarction; T2D, type 2 diabetes; HDL-C, high density lipoprotein cholesterol; LDL-C, low density lipoprotein cholesterol; TC, total cholesterol; TG, triglycerides; FG, fasting glucose.
